# Gender Differences in Core Muscle Morphology of Elite Alpine Skiers: Insights from Ultrasonography

**DOI:** 10.3390/s24134073

**Published:** 2024-06-22

**Authors:** Carlos Romero-Morales, Jorge H. Villafañe, Rafael Jácome-López, Marco Tullio, Agustín Strelczuk, Diego Miñambres-Martín, José Luis Lara-Cabrero, Sergio L. Jiménez-Sáiz

**Affiliations:** 1 Faculty of Sport Sciences, Universidad Europea de Madrid, 28670 Villaviciosa de Odón, Spain; carlos.romero@universidadeuropea.es (C.R.-M.); mail@villafane.it (J.H.V.); diego.minambres@universidadeuropea.es (D.M.-M.); joseluis.lara@clinicacemtro.com (J.L.L.-C.); 2 GlobalPhysio Rehabilitation Clinic, 28010 Madrid, Spain; rjacome@globalphysio.es (R.J.-L.); marco00tullio@gmail.com (M.T.); strelczukagustin@gmail.com (A.S.); 3 Clinica CEMTRO, 28035 Madrid, Spain; 4 Sport Sciences Research Centre, Universidad Rey Juan Carlos, 28942 Fuenlabrada, Spain

**Keywords:** performance assessment, lower limb muscles, ultrasonography, elite skiers

## Abstract

This study investigates gender differences in core muscle morphology among elite alpine skiers using ultrasonography, highlighting significant disparities that could influence training and injury prevention strategies. Methods: A cross-sectional design was employed, examining ultrasound imaging (USI) in 22 elite skiers (11 male, 11 female) to assess the thickness of the external oblique (EO), internal oblique (IO), transversus abdominis (TrAb), and rectus abdominis (RA) muscles. Results: Significant differences were noted, with male skiers displaying greater muscle thickness, particularly in the right IO and RA and left IO, EO, TrAb, and RA. Conclusions: These findings suggest that male and female skiers may require different training approaches to optimize performance and reduce injury risks. This research contributes to a deeper understanding of the physical demands on elite skiers and underscores the need for gender-specific training regimens to enhance athletic outcomes and prevent injuries.

## 1. Introduction

Assessment of core muscles, such as abdominal wall and trunk muscles, plays a critical role in sports science, offering vital insights into the core stability and athletic performance of elite athletes [1,2]. Elite alpine skiing has been associated with overuse or traumatic injuries, and the low back area has been considered the most affected body region in young elite alpine skiers [3]. Core stability is pivotal for success in skiing, a sport that demands high levels of balance, agility, and muscular coordination [4,5]. The role of the core muscles in maintaining stability has been demonstrated in explosive and high-demand tasks, such as arm and leg movements, walking and running [6], and lifting and landing [7]. During skiing activities, the lumbar spine and the abdominal wall muscles—external oblique (EO), internal oblique (IO), transversus abdominis (TrAb), and rectus anterior (RA)—endure challenging conditions, which can lead to the development of overuse injuries. These conditions include motions such as spine flexion, lateral flexion, and rotation, all compounded by high levels of vibration [8]. Prior research showed that young skiers may experience various overuse-related structural changes in the core muscle system, some of which are significant enough to limit participation in elite sports [9]. In this context, adult elite skiers reported that overuse-related back issues are prevalent not just during the competitive season but also throughout the preparatory stage, which includes both off-snow and snow training [10].

The structural integrity of the abdominal wall not only supports athletic performance but also acts as a preventive measure against injuries. It is increasingly acknowledged that the morphology and functionality of the abdominal muscles may differ significantly between genders, influencing training and rehabilitation programs. Core muscles work in a coordinated manner with the pelvic floor muscles and diaphragm to pressurize the abdominal cavity and transfer mechanical loads from the lower limb to the trunk [11]. Several authors reported that functional and structural conditions of the abdominal wall muscles were related to lumbopelvic pain and chronic low back pain [12,13]. In this context, gender differences were observed in these muscle complexes, such as different muscle activation strategies during landings [7], or even females choosing to land in a more erect posture to maximize energy absorption from the joints most proximal to ground contact [14]. For example, these trunk muscle activation characteristics have been shown to be sex-specific: in preparing for a sudden trunk load, females augment the RA and EO muscles more than males [15].

In recent years, ultrasonography has emerged as a preferred method for examining muscle characteristics due to its non-invasive nature, reliability, cost-effectiveness, and capacity for real-time analysis [16,17,18]. This technique has been extensively validated and is considered highly reliable for measuring muscle thickness and other morphological features of the abdominal muscles, such as the TrAb, RA, IO, and EO [19]. Additionally, all these examinations can be performed in a reliable and valid way in static and dynamic B-Mode [1,11]. Previous studies have leveraged ultrasonography to explore muscle adaptations across various sports disciplines, revealing crucial sex-specific differences that could influence the approach to training and recovery [1,20].

However, few studies have specifically explored these muscular characteristics among elite skiers, despite the high demands placed on their core muscles during both training and competitive events [21]. The dynamic and physically demanding nature of skiing, characterized by intense lateral movements and balance maintenance, underscores the importance of a robust and responsive core [22]. Furthermore, understanding the sex-specific muscular architecture in this group could lead to more tailored and effective training regimens, enhancing performance while reducing the risk of injury.

Therefore, this study employed USI to compare and quantify EO, IO, TrAb, and RA muscle thickness and the IRD between elite male and female skiers. We hypothesized that, despite male and female elite alpine skiers undergoing similar training sessions and workout programs, the thickness of the abdominal wall muscles would be greater in men. This approach aims to enhance our understanding of gender disparities in core muscle morphology within elite athletic settings, potentially informing the development of training and rehabilitation protocols tailored for winter sports.

## 2. Materials and Methods

### 2.1. Study Design

A cross-sectional study was conducted to assess differences in the thickness of the abdominal wall muscles between elite male and female skiers, following the Strengthening the Reporting of Observational Studies in Epidemiology (STROBE) [23] criteria, from February to March 2024. Previously, the Research and Ethics Committee of Universidad Europea de Madrid (Madrid, Spain) approved the study. Before starting this research, an informed consent form was read and signed by all participants. In addition, the ethical recommendations and considerations of the Helsinki Declaration were respected during the course of this study [24].

### 2.2. Participants

A sample of 22 elite skiers were recruited and divided into a male group (*n* = 11) and a female group (*n* = 11). Elite alpine skiers are considered those who ski more than 180 days a year, participate in national and international competitions, are sponsored by their respective federations to attend training camps at high-performance centers, and compete at both national and international levels. They all participate during the same year in four main types of races: four slalom races, four giant slalom races, one super giant slalom race, and one downhill race. Inclusion criteria were as follows: age from 16 to 21 years, elite skiers, and able to perform the training sessions. Exclusion criteria were as follows: previous abdominal surgery or abdominal hernia, rheumatological conditions, tissue disturbances, systemic diseases, neurological or musculoskeletal disorders, and orthopedic surgical procedures in the lumbar region, pelvis, or lower limbs during the previous 6 months. In addition, allergic reaction to the ultrasound gel was considered an exclusion criterion.

### 2.3. Outcome Measurements

**Figure 1:**
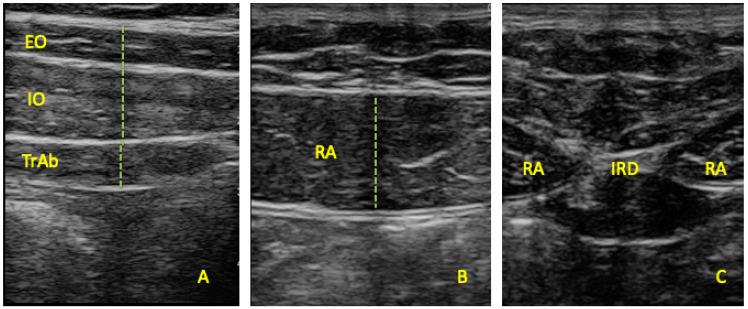
Ultrasound thickness assessments of the (**A**) EO, IO, TrAb, (**B**) RA, and (**C**) IRD.

All assessments were conducted by the same clinician (M.T.), who is experienced in using ultrasound imaging. Ultrasound examinations were performed employing a SonoScape E2 (SonoScape Equipment, Madrid, Spain) equipped with a linear transducer ranging from 8 to 13 MHz and with a 55 mm footprint, operating in B-Mode. Participants lay in a supine position during the collection of ultrasound data. Adhering to the protocols set forth by Whittaker et al. [25], imaging of the EO, IO, and TrAb muscles was carried out by positioning the transducer along the mid-axillary line, equidistant from the subcostal line to the iliac crest (Figure 1A). For evaluating the RA, the transducer was aligned with the umbilicus and positioned just below it for measuring the inter-recti distance (IRD) (Figure 1B). According to Whittaker et al., the IRD is defined as the linear gap between the rectus abdominis muscles on either side (Figure 1C) [25]. Images were recorded, showing excellent intra- and inter-rater reliability (ICC = 0.92–0.99) [26]. Muscle thickness was quantified by measuring the distance across the muscle borders. All measurements were evaluated on both sides.

### 2.4. Statistical Analysis

Analyses were performed using RStudio (v.1.4, RStudio, PBC, Boston, MA, USA) and Jamovi (v.1.6, The Jamovi Project). Normality assumption was checked by the Shapiro–Wilk test (*p* > 0.05). Descriptive data were described using mean and standard deviation (SD) for parametric data and median and interquartile range (IR) for non-parametric data. For the comparative analysis of parametric data for both groups, the Student’s *t*-test for independent samples was used, whereas for non-parametric data analysis, the Mann–Whitney U test was used. Moreover, Levene’s test was used to check the homogeneity of variances. The effect size between groups was estimated using Cohen’s d, interpreting values of 0.2 as small, 0.5 as medium, and 0.8 as large effects.

## 3. Results

The present study aimed to investigate gender differences in the core muscle morphology of elite alpine skiers using ultrasonography. The analysis revealed significant disparities in muscle thickness between male and female skiers, which are detailed below.

### Sociodemographic Characteristics

The sample comprised 22 elite alpine skiers, divided equally between males and females. The demographic analysis indicated significant differences between genders concerning weight, height, and BMI. Specifically, male skiers exhibited higher mean values in these parameters. The average weight for male skiers was significantly higher at 74.2 ± 6.2 kg compared to 61.9 ± 6.9 kg for female skiers (*p* = 0.002). Similarly, male skiers were taller, with an average height of 1.75 ± 3.9 cm compared to 1.67 ± 0.7 cm for females (*p* = 0.005). The BMI was also greater in male skiers (23.7 ± 1.5 kg/m^2^) than in female skiers (22.0 ± 1.54 kg/m^2^, *p* = 0.024) (Table 1).

Considering Table 2, the ultrasonographic assessment focused on measuring the thickness of key abdominal muscles, including the EO, IO, TrAb, and RA on both sides. The results demonstrated that male skiers generally have thicker core muscles than female skiers. **Right-side muscles**: Male skiers showed significantly greater muscle thickness in the right IO and RA muscles. The right IO muscle thickness was 12.6 ± 2.69 mm in males compared to 9.04 ± 1.78 mm in females (*p* = 0.001). The right RA muscle was also thicker in males (16.4 ± 2.22 mm) than in females (12.7 ± 1.46 mm, *p* = 0.001). **Left-side muscles**: Similar significant differences were observed on the left side, where males exhibited greater thickness in the IO, EO, TrAb, and RA muscles. For instance, the left IO muscle thickness was 8.53 ± 1.96 mm in males and 8.29 ± 1.14 mm in females (*p* = 0.001). The left EO and RA muscles also showed higher thickness values in males (EO: 8.53 ± 1.96 mm vs. 8.29 ± 1.14 mm, *p* = 0.001; RA: 16.1 ± 2.16 mm vs. 12.2 ± 1.20 mm, *p* = 0.001). Additionally, the left TrAb muscle was significantly thicker in males (4.45 ± 1.25 mm) compared to females (3.36 ± 0.8 mm, *p* = 0.028).

These results indicate that male skiers possess more developed core muscles, which may be attributed to the differing physical demands and muscle activation patterns experienced by each gender during high-intensity skiing activities. The greater muscle thickness observed in males is likely a response to the higher load-bearing and stability requirements typical in male-dominated skiing disciplines.

## 4. Discussion

In terms of the current knowledge, this study is the first detailing novel insights into the morphological features of core muscles in elite alpine skiers, highlighting significant sex-specific differences that have important implications for athletic training and injury prevention. These findings suggest that, despite similar training regimens, male skiers develop thicker core muscles. This disparity could be attributed to inherent physiological differences, such as higher testosterone levels in males, which promote greater muscle hypertrophy. Additionally, the higher physical demands and load-bearing activities in male alpine skiing might contribute to the observed differences in muscle thickness.

Consistent with prior research on athletes from various sports disciplines, our findings show marked differences in the muscle thickness of the EO, IO, TrAb, and RA between young elite male and female skiers. These differences could be explained by adaptations to the distinct physical demands faced by each gender in elite skiing, which is characterized by intensity and dynamic actions requiring robust core stability and strength. Holmberg et al. support that the upper-body musculature plays a key role in the high-demand actions of elite skiers, who must be prepared for the high demands and muscle activations required [27]. The greater muscle thickness observed in male skiers may be attributed to the higher physical demands and load-bearing activities typically encountered in male-dominated sports, which necessitate greater muscular support to maintain spinal stability and distribute mechanical loads effectively. This aligns with the findings from Spörri et al., who noted that back injuries in skiing are often the result of complex biomechanical patterns involving high-impact and torsional forces, necessitating strong and responsive core muscles to prevent injury [28]. Furthermore, the significance of core muscle morphology in preventing overuse injuries, specifically in the lumbar region, has been well documented in the literature. As noted by Frohlich et al., young skiers experience various overuse-related structural changes that can limit participation, emphasizing the need for targeted preventive measures. Moreover, discipline differences, previous traumatic injuries, and overall training load may play key roles in lower limb and back musculoskeletal condition manifestations [29]. Our results suggest that tailored training and rehabilitation programs that account for these morphological differences between genders could enhance performance and reduce the incidence of injuries among elite skiers.

The literature has documented gender differences during athletic maneuvers such as running and cutting, due to researchers better understanding the biomechanical mechanisms related to athletic injuries [30,31,32]. Considering gender differences in anatomy and muscle activation, Da Cuña-Carrera showed that standing TrAb and EO increased during hypopressive exercises, with higher effects in men, which could be explained by differences in anatomy and position between sexes [33]. These differences are crucial for optimizing performance and reducing injury risks, as evidenced by lower rates of core and lower back injuries in sports where gender-specific training protocols are implemented.

The use of ultrasonography in our study also underscores the utility of this non-invasive technique in providing real-time, accurate assessments of muscle morphology. This capability is crucial for designing rehabilitation protocols that are responsive to the specific needs of athletes, as highlighted by Romero et al., who demonstrated ultrasound’s effectiveness in identifying subtle changes in muscle structure that are relevant for clinical interventions [1,2]. In elite sports, the assessment of the muscle anatomical and mechanical properties has a great potential for testing athletes’ response to training, detecting yellow and red flags in the injury prevention context, and monitoring their return to sport after a muscle injury [34].

## 5. Limitations and Future Directions

This study is not without limitations. The sample size, although sufficient for initial findings, is relatively small and limited to elite skiers. This restricts the generalizability of the results to the broader population of recreational or amateur skiers. Furthermore, the cross-sectional design of the study provides a snapshot in time and does not account for the dynamic changes in muscle morphology that occur throughout a training or competitive season. The study’s focus on core muscles excludes other critical muscle groups involved in skiing, such as the leg and hip muscles. Finally, the influence of environmental factors, such as altitude and temperature, which can affect muscle performance and injury risk, was not considered in this study. Future research should take these variables into account to fully understand their impact on muscle function and performance in alpine skiing environments.

The findings of this study pave the way for several avenues of future research. First, longitudinal studies are needed to examine the changes in core muscle morphology over multiple seasons or competitive cycles. Such studies could offer insights into how training regimens influence muscle adaptations longitudinally and help identify the optimal timing and type of interventions to prevent injuries and enhance performance. Additionally, exploring the effects of specific core-strengthening programs on muscle morphology using ultrasonography could validate the direct impact of tailored exercise regimes on abdominal muscle performance in skiing.

Moreover, the inclusion of a broader range of athletes from various skiing disciplines could provide a more comprehensive view of the sport-specific demands on muscle architecture. Comparative studies with athletes from other winter sports, such as snowboarding or biathlon, may also uncover unique patterns of muscle use and adaptations that could inform cross-disciplinary training strategies. Finally, integrating biomechanical data, such as force and motion analysis, with ultrasonographic findings could enhance the understanding of how muscle characteristics relate to actual performance and biomechanical outputs in skiing.

## 6. Practical Applications

The findings of the present study have substantial clinical implications for sports medicine, rehabilitation, and athletic training. Male skiers, with their thicker core muscles, may have an advantage in stabilizing the spine and absorbing mechanical loads during skiing, thereby reducing the risk of overuse injuries. However, the thinner core muscles in female skiers suggest a potential vulnerability to such injuries. For female skiers, whose core muscles are typically thinner, targeted core-strengthening exercises aimed at increasing muscle mass and stability are essential. Examples include high-repetition core exercises, resistance band workouts, and stability ball exercises. To address the higher potential vulnerability to overuse injuries in female skiers, injury prevention protocols should include exercises that enhance core stability and improve neuromuscular coordination. This can involve proprioceptive training, balance exercises, and functional movement drills. For male skiers, injury prevention can focus on maintaining the strength and flexibility of the core muscles to handle the high physical demands of skiing. Additionally, rehabilitation programs for injured skiers should be designed to address the specific weaknesses identified in core muscle morphology. For male skiers, rehabilitation should include exercises that maintain muscle hypertrophy and prevent atrophy during recovery, such as progressive resistance training and isometric core exercises. For female skiers, rehabilitation should focus on exercises that enhance muscle activation and stability, ensuring a gradual and safe return to full activity. Techniques such as neuromuscular re-education and core-stabilization exercises can be beneficial. By adopting these tailored approaches, coaches and clinicians can optimize the performance of elite alpine skiers while reducing the risk of core-related injuries. These recommendations underscore the importance of considering gender-specific differences in muscle morphology when designing and implementing training and rehabilitation protocols.

## 7. Conclusions

Gender-specific differences were found for the thickness of the right IO and RA and left TrAb, IO, EO, and RA, which were thicker in elite male skiers with respect to elite female skiers, as assessed by USI. These findings suggest that tailored training and rehabilitation programs are necessary to optimize performance and reduce injury risks for each gender. Male skiers benefit from high-intensity core exercises to maintain muscle endurance and stability, while female skiers require targeted core strengthening to increase muscle mass and stability. Regular ultrasonographic assessments are essential to monitor and adjust training programs effectively. These insights highlight the importance of gender-specific training protocols in sports medicine and athletic training, contributing to enhanced performance and injury prevention for elite skiers. Implementing these recommendations can significantly improve the health and career longevity of athletes.

## Figures and Tables

**Table 1:** Sociodemographic data of the sample.

Measurement	Female (*n* = 11) Mean ± SD	Male (*n* = 11) Mean ± SD	*p*-Value
Age, y	17.3 ± 1.35	16.6 ± 0.5	0.177
Weight, kg	61.9 ± 6.9	74.2 ± 6.2	0.002
Height, cm	1.67 ± 0.7	1.75 ± 3.9	0.005
BMI, kg/cm^2^	22.0 ± 1.54	23.7 ± 1.5	0.024

**Table 2:** Ultrasound imaging measurements of the abdominal wall muscles.

Measurement	Female (*n* = 11) Mean ± SD (95% CI)	Male (*n* = 11) Mean ± SD (95% CI)	*p*-Value (Effect Size)
TrAb_R	3.47 ± 0.7 (2.3–5.1) *	4.04 ± 0.9 (2.4–5.5) *	0.128 (0.67) **
IO_R	9.04 ± 1.78 (6.4–12.6) *	12.6 ± 2.69 (8.8–17.1) *	0.001 (1.57) **
EO_R	7.88 ± 1.21 (5.9–9.8) *	8.53 ± 1.56 (5.7–12.1) *	0.356 (0.40) **
RA_R	12.7 ± 1.46 (10.8–15.0) *	16.4 ± 2.22 (13.2–20.7) *	0.001 (1.90) **
TrAb_L	3.36 ± 0.8 (2.7–8.3) ^†^	4.45 ± 1.25 (2.70–7.21) *	0.028 (0.80) ^‡^
IO_L	8.29 ± 1.14 (6.2–9.6) *	8.53 ± 1.96 (5.7–12.1) *	0.001 (3.08) **
EO_L	8.29 ± 1.14 (5.4–9.2) *	8.53 ± 1.96 (7.1–12.0) *	0.001 (1.57) **
RA_L	12.2 ± 1.20 (10.3–14.9) *	16.1 ± 2.16 (12.0–19.1) *	0.001 (2.26) **
IRD	6.85 ± 2.38 (3.9–11.6) *	7.80 ± 3.14 (3.1–14.7) *	0.431 (0.34) **

Abbreviations: EO, external oblique; IO, internal oblique; IRD, inter-recti distance; RA, rectus abdominis; TrAb, transversus abdominis; L, left; R, right. * Mean (SD) was applied. ** Student’s *t*-test for independent samples was performed. ^†^ Median (IR) was used. ^‡^ Mann–Whitney *U* test was utilized.

## Data Availability

The original contributions presented in the study are included in the article, further inquiries can be directed to the corresponding author.
